# A Scale-Free Structure Prior for Graphical Models with Applications in Functional Genomics

**DOI:** 10.1371/journal.pone.0013580

**Published:** 2010-11-05

**Authors:** Paul Sheridan, Takeshi Kamimura, Hidetoshi Shimodaira

**Affiliations:** Department of Mathematical and Computing Sciences, Tokyo Institute of Technology, Tokyo, Japan; Johns Hopkins University, United States of America

## Abstract

The problem of reconstructing large-scale, gene regulatory networks from gene expression data has garnered considerable attention in bioinformatics over the past decade with the graphical modeling paradigm having emerged as a popular framework for inference. Analysis in a full Bayesian setting is contingent upon the assignment of a so-called structure prior—a probability distribution on networks, encoding *a priori* biological knowledge either in the form of supplemental data or high-level topological features. A key topological consideration is that a wide range of cellular networks are approximately scale-free, meaning that the fraction, 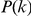, of nodes in a network with degree 
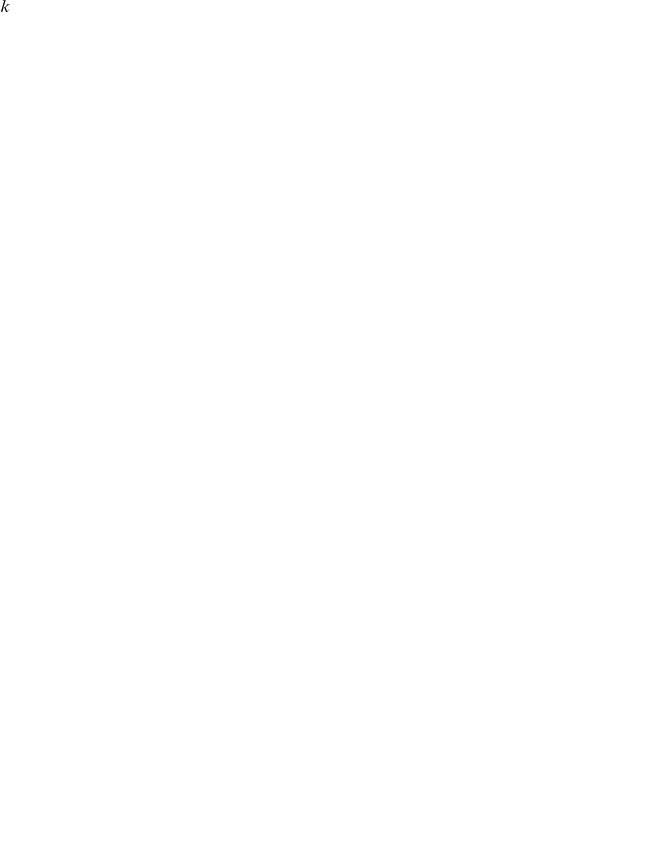
 is roughly described by a power-law 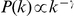 with exponent 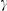 between 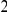 and 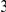. The standard practice, however, is to utilize a random structure prior, which favors networks with binomially distributed degree distributions. In this paper, we introduce a scale-free structure prior for graphical models based on the formula for the probability of a network under a simple scale-free network model. Unlike the random structure prior, its scale-free counterpart requires a node labeling as a parameter. In order to use this prior for large-scale network inference, we design a novel Metropolis-Hastings sampler for graphical models that includes a node labeling as a state space variable. In a simulation study, we demonstrate that the scale-free structure prior outperforms the random structure prior at recovering scale-free networks while at the same time retains the ability to recover random networks. We then estimate a gene association network from gene expression data taken from a breast cancer tumor study, showing that scale-free structure prior recovers hubs, including the previously unknown hub SLC39A6, which is a zinc transporter that has been implicated with the spread of breast cancer to the lymph nodes. Our analysis of the breast cancer expression data underscores the value of the scale-free structure prior as an instrument to aid in the identification of candidate hub genes with the potential to direct the hypotheses of molecular biologists, and thus drive future experiments.

## Introduction

### Gene Regulatory Networks and Gene Expression Data

Knowledge of the interactions among genes and gene products that occur within a cell is vital for understanding cellular behavior. These activities are largely a consequence of gene expression, the process whereby genes transcribe signature mRNA molecules that are translated into gene products of numerous kinds and functions. As it happens, genes do not express independently of one another; instead, their activities are coordinated in a complex system of control in which distinguished genes, called transcriptions factors, regulate the expression of other genes via their gene product proxies.

An undirected network 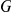 is a mathematical object consisting of a set of nodes and a set of unordered pairs of nodes called undirected edges. It differs from a directed network, which is also denoted by 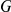, in that the latter is defined in terms of ordered pairs of nodes known as directed edges. Applying these straightforward abstractions to cellular processes has gained currency throughout the biosciences, so much so that a network mind-set has become a necessary precondition for thinking about systems of gene regulatory interactions. For the purposes of this paper, a gene regulatory network is a directed network in which genes are identified with nodes and regulatory interactions with directed edges. From a purely statistical standpoint, it is best to regard a gene regulatory network as a convenient depiction of the true regulatory interactions of a system that, in reality, must be estimated from data.

Indeed, the network approach toward understanding gene regulatory systems only came to prominence in response to the advent of DNA microarray technology, which makes the profiling of mRNA expression levels for individual genes possible on a genome-wide scale. A typical experiment consists of a library of 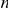 expression profiles, each one a snapshot of the expression levels for 
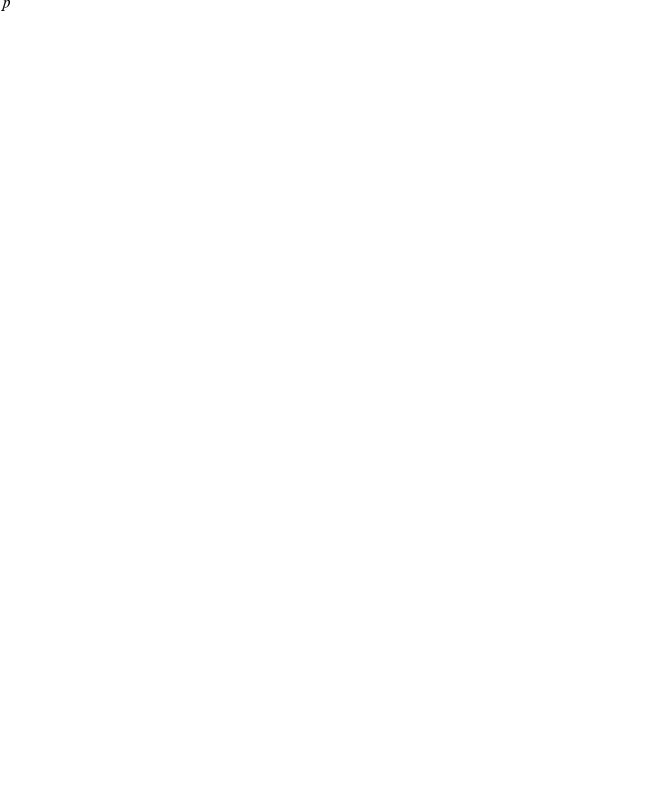
 genes under a different experimental condition. The raw expression profile data is preprocessed and then arranged by row in an 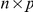 data matrix, 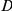. In practice, not only is gene expression data notoriously noisy [1], but to make matters worse the number of samples is typically at least an order of magnitude smaller than the number of genes, that is, 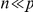 (the “small 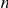, large 
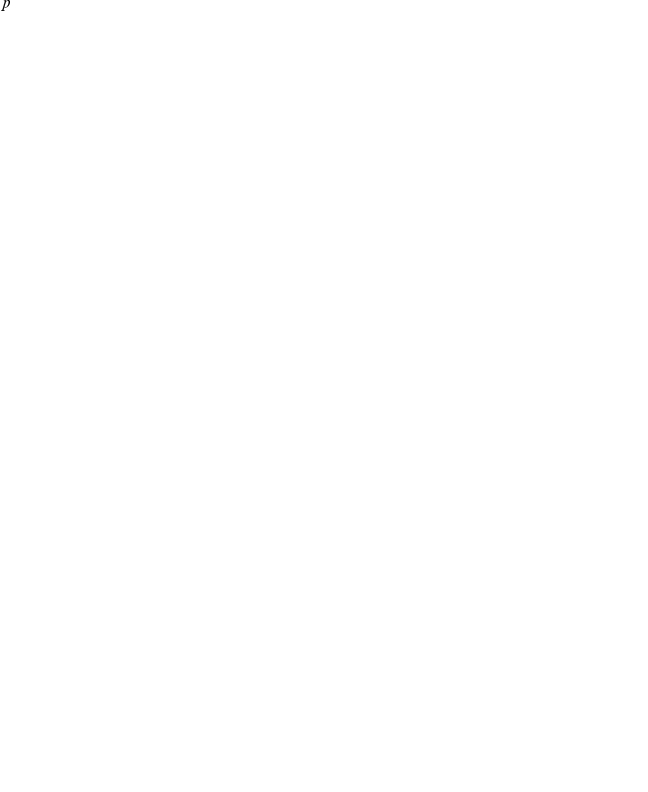
” problem), making the inference of regulatory interactions a challenging statistical problem [2].

There is now an extensive repertoire of algorithms available for the analysis of gene expression data, the majority of which are based on Boolean networks, differential equations, and graphical models [3]. Some approaches produce estimated gene regulatory networks that are directed networks, while others do not. In this paper, we work primarily with a variety of undirected graphical model known as gene association networks (GANs), in which undirected edges, called gene associations, correspond to certain statistical dependencies that are inferred from gene expression data. Therefore, in an effort to simplify the terminology, the terms “network” and “edge” will be used hereafter to mean undirected network and undirected edge, respectively. Although we will occasionally use the term “network” in a colloquial sense, such as in “network mind-set” or “network approach.” At any rate, the meaning should be clear from context.

### Graphical Models

Graphical models [4], [5], [6] are a suite of probabilistic models, widely used for estimating large-scale gene regulatory networks from gene expression data [7]. In this framework, genes are identified with the random variables of a multivariate distribution 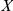 with covariance matrix 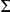, and each row of 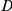 is taken as a random sample from 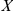. The conditional independence structure of 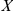 defines a network with the random variables as nodes and conditional dependencies latent between the random variables as directed or undirected edges; a diversity of models arise from the extent to which the dependencies are resolved [8].

Relevance networks comprise the simplest class of graphical model with absent edges corresponding to marginal independencies between the components of 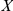. These networks have long been used in the analysis of genetic data [9]. But in terms of identifying regulatory interactions, relevance networks are bound to be misleading because marginal independence alone cannot discriminate among direct and indirect dependencies.

GANs provide a better alternative, circumventing this drawback by appealing to conditional independence as a criterion for edge exclusion. Gaussian graphical models (GGMs) are the gold standard. In a GGM, a pair of nodes do not share an edge when their underlying random variables from 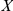 are conditionally independent given all of the remaining random variables. However, GGMs too are not without disadvantages, as their estimation can be computationally intensive in a “small 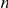, large 
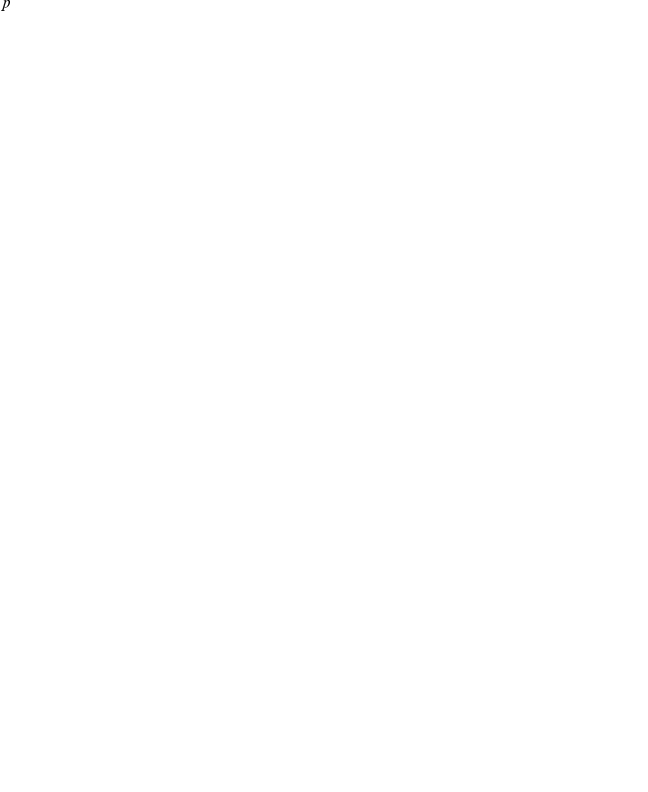
” setting [10]. A class of GANs, bridging the gap between relevance networks and GGMs, has been advanced with this consideration in mind where absent edges are identified with lower order conditional independencies [11], [12], [13].

Lastly, Bayesian networks are a variety of graphical model founded on a more refined notion of conditional independence, conferring directionality to the edges; they are also well-established as a methodology for estimating gene regulatory networks [14].

### The Structure Prior

Inference within the graphical modeling paradigm amounts to an often painstaking exercise in covariance estimation and model selection. We defer a discussion on the problem of covariance estimation to the Methods section. That is because our interest pertains to model selection, which in a Bayesian setting is accomplished by sampling from the posterior distribution
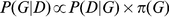
(1)over the appropriate space of networks using either heuristic searches or else Markov chain Monte Carlo (MCMC). The term 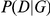 is the likelihood and 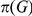 the structure prior, that is, a prior assigning a probability to each possible network.

The role of the structure prior is to direct inference toward graphical models consistent with biological prior knowledge, which may come in the form of *a priori* topological considerations or from *a posteriori* sources apart from the dataset. As far as the latter is concerned, previous research has concentrated on Bayesian networks [15], [16], [17], [18]. On the other hand, biologically-motivated topological assumptions are a consistent feature of graphical models tailored for genetic data. Heuristic search strategies often include implicit assumptions concerning network sparsity [19], [20], [21], [22]. In instances in which the structure prior is given explicit specification, standard practices include using a uniform prior capped at a small number of potential regulators per gene [23], or assigning it as a sparse random network [24], [25].

### Random and Scale-Free Networks

The theory of random networks was given its first systematic expression by Erdös and Rényi [26], [27]. According to the theory, a 
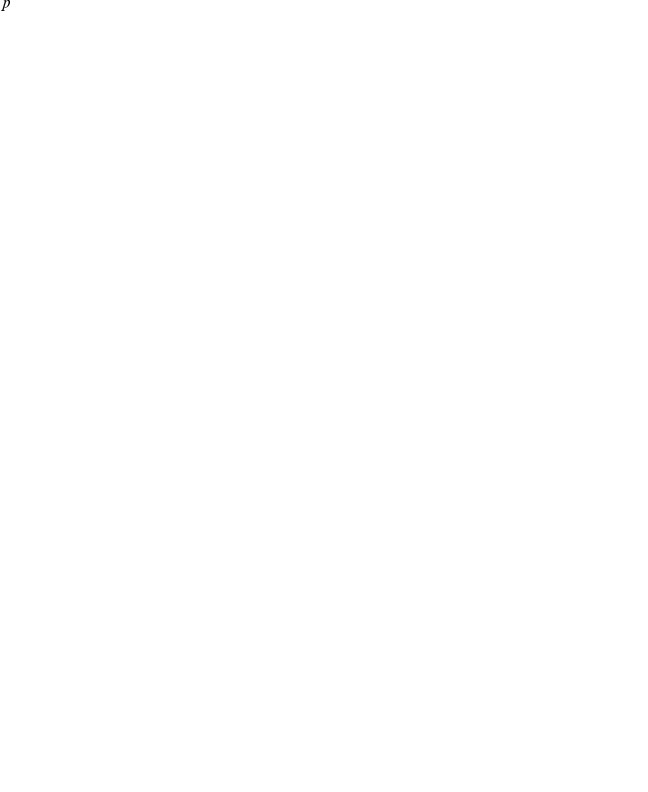
-node random network is defined by an eponymous, generating algorithm — the Erdös-Rényi (ER) model — that works by connecting each pair of nodes in an initially empty network independently with probability 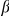. This simple procedure gives rise to a probability distribution over the space of 
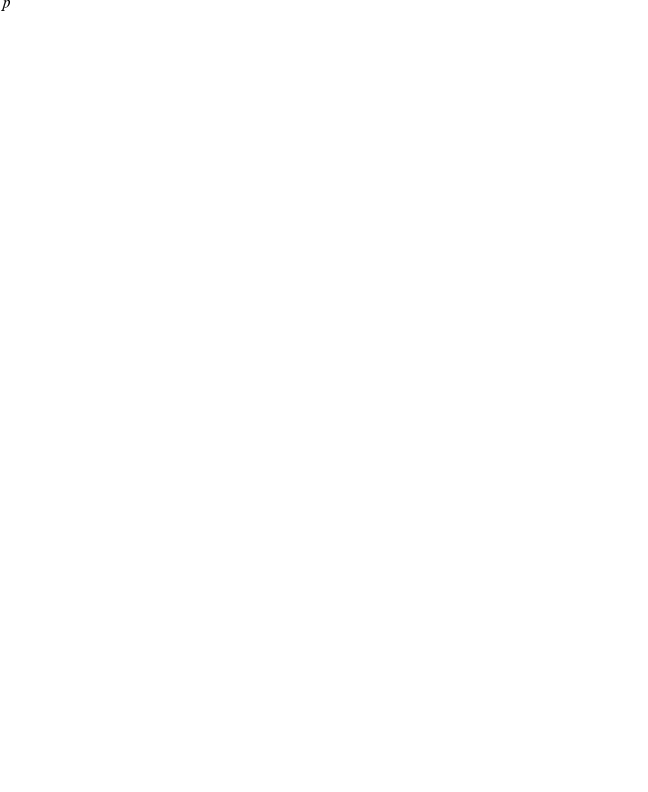
-node networks, which is used to define the so-called random structure prior, 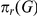. The degree distribution 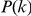 — the probability that a given node is connected to 
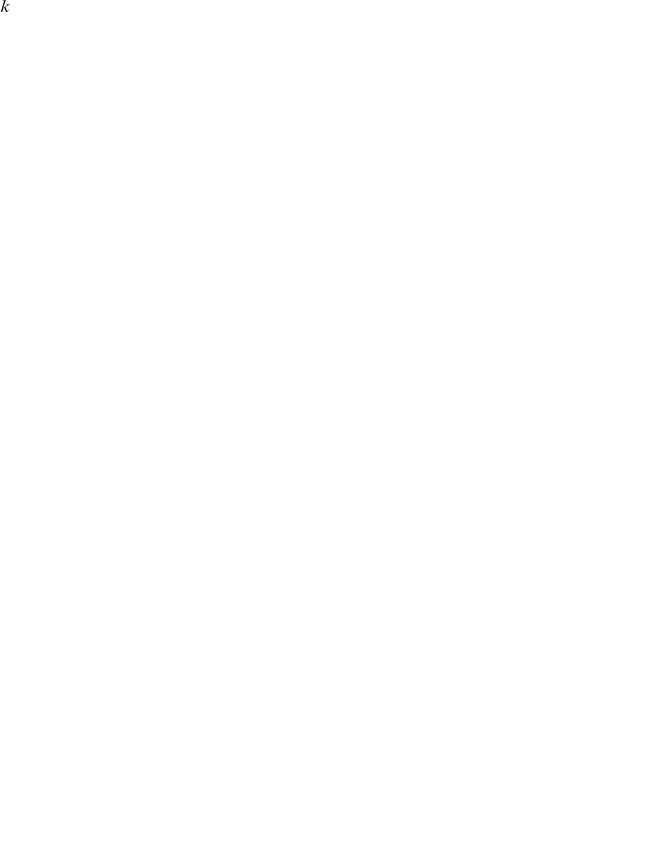
 other nodes — of a random network is binomially distributed according to 
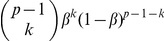
 where the degree, 
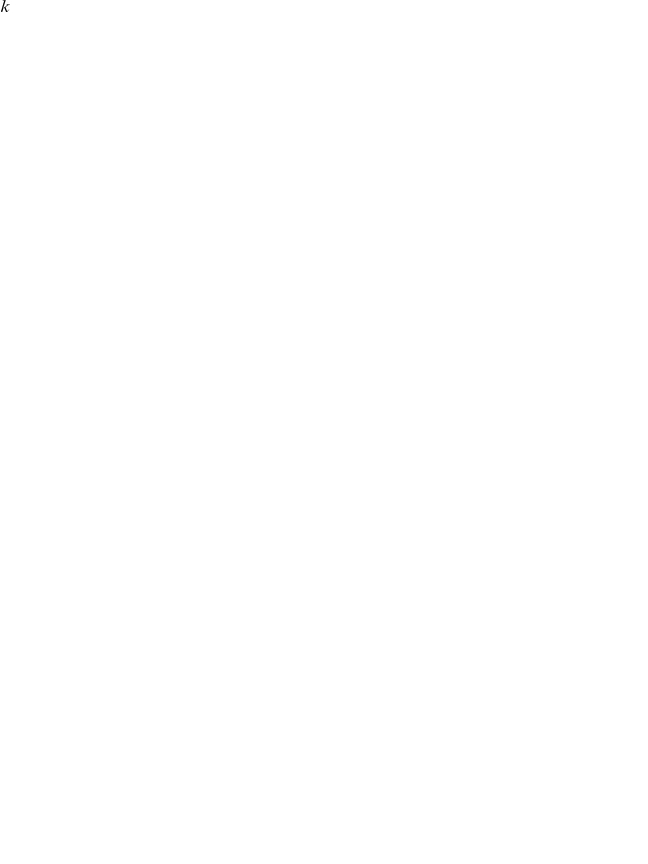
, of a node denotes the number of edges incident upon it. It follows, therefore, that degree in a random network has a strong central tendency, implying that the average degree of a random network is representative of the degree of a typical node.

Empirical studies, however, have firmly established that a wide variety of large-scale networks in nature, society, and technology exhibit heavy-tailed degree distributions that cannot be accounted for by random network theory [28], [29], [30]. This property is often approximately described by a power-law degree distribution, 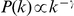, over a large range of 
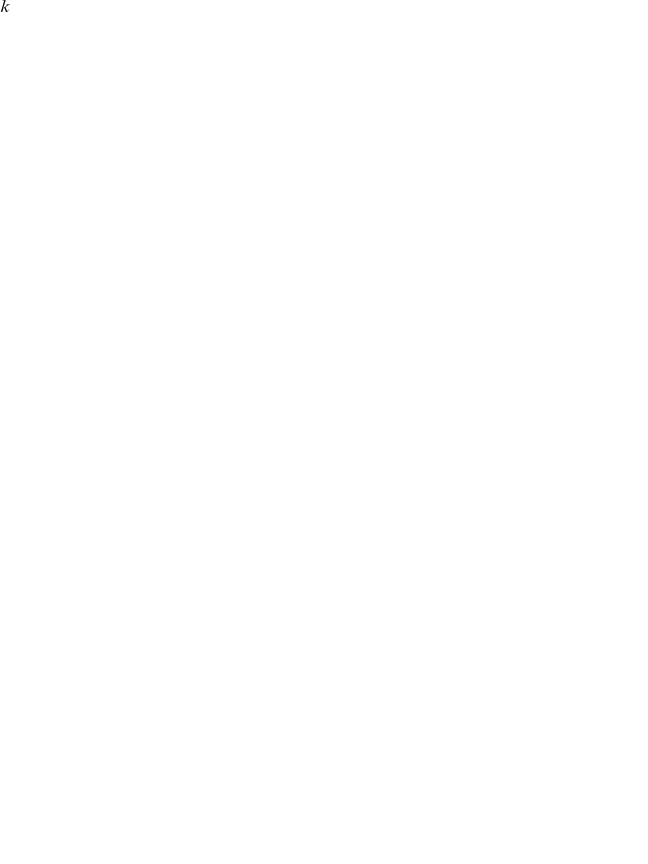
 with exponent 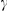 typically between 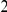 and 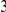. A network that follows a power-law is called scale-free. It gets this name because the functional form of 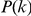 is retained under a scaling of the argument 
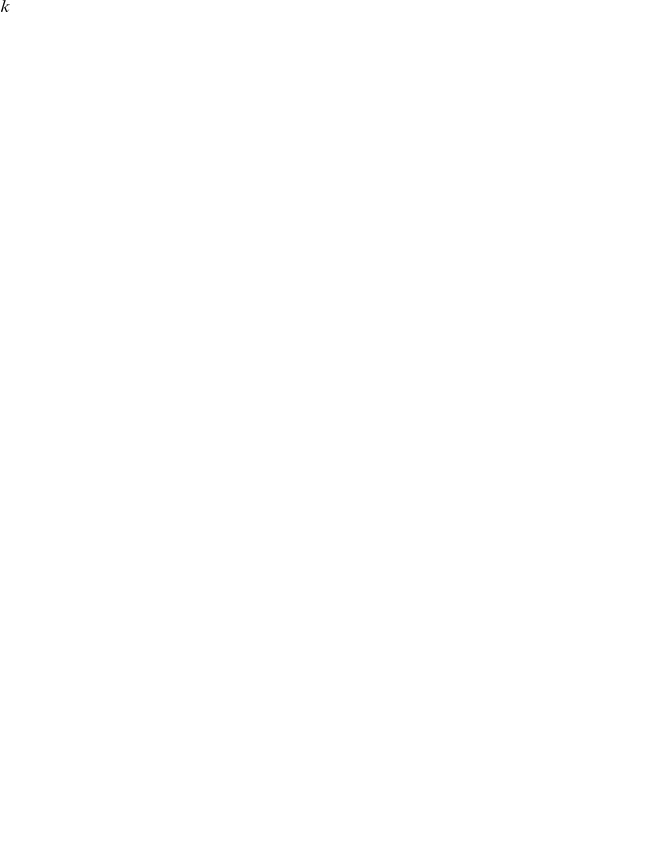
 by a constant factor 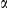: 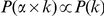. The scale-free property is thought to be a key organizational feature of cellular networks [31], and analyses suggest such an architecture for the gene regulatory networks of the model organisms *S. cerevisiae* [32] and *C. elegans* [33].

### Introducing a Scale-Free Structure Prior

Proposing a structure prior which incorporates the scale-free property is the topic of this paper. We define the scale-free structure prior, 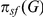, according to the probability of a network under a simple, scale-free network model. As for the underlying network model itself, a multitude of candidates have been proposed in the literature [34]. They fall into two broad categories: 1) growing models, where a network is generated via the successive addition of nodes and edges to a small seed network, and 2) non-growing models, where to a fixed number of nodes, pairs of nodes are chosen randomly and connected by edges.

The growing model approach employs a handful of simple universal mechanisms, thought to underpin disparate natural phenomena, to drive the stochastic evolution of networks toward power-laws. Preferential attachment is, perhaps, the best known mechanism. The idea works something like this: the probability of attaching an edge from a newly added node to a node already in the system is roughly proportional to the degree of the old node. The Bárabasi-Albert (BA) model [35] is the latter-day progenitor of a wide variety of preferential attachment models. The BA model generates a network via the successive addition of nodes and edges to a small seed network. At each step, a node is added to the system with a fixed number of emanating edges, which are subsequently preferential attached to the existing nodes. The resulting network follows a power-law with 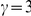 on average.

Preferential attachment is not considered to be the main driving force behind genome evolution; instead, gene (node) duplication and point mutations (edge dynamics) play the dominant role in shaping of gene regulatory networks [36]. The duplication model as formulated by [37] is such a network model, which in an analysis by [38] is suggested to approximately follow a power-law.

By contrast, in the non-growing approach, each node is assigned a fixed weight with the probability of a particular network depending on those weights. The ER model is an example of a non-growing model with uniform weights. Another non-growing model is the static model [39], which is a generalization of the ER model that has been shown to follow a power-law with 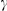 tunable to any value greater than 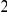, depending on the specification of the model parameters; see Methods for details. We use the static model to define 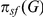. Indeed, this model is an appealing candidate for the purpose as the probability of a network is easy to compute compared with growing models of similar complexity. Moreover, the static model actually includes the ER model as a limiting case.

### A New Metropolis-Hastings Sampler for Networks

We implement an MCMC algorithm with 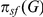 for GGMs adapted from [25], although it is important to point out that our methodology applies to graphical models in general. Reworking the algorithm is not simply a matter of plugging in a formula for 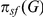 because it depends furthermore on a labeling of the nodes of 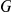. Confronted with this complication, we design a novel Metropolis-Hastings sampler that solves the problem by including a node labeling, 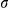, which is defined in the Methods section, as a variable in the state space, thereby allowing it to be estimated.

### Summary of Contributions

In this paper, we advance a scale-free structure prior, 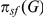, for graphical models defined by the formula for the probability of a network under the static model. Our objective is to compare the performance of this prior with the commonly used random structure prior, 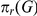, in the arena of simulation as well as with a real data example. We choose GGMs for this purpose, modifying the MCMC algorithm of [25] to include 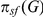. As mentioned above, one challenge of implementing 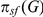 is that, unlike with 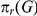, it requires a labeling of the nodes of 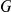. We address this issue by introducing a Metropolis-Hastings sampler that includes the node labeling as a variable in the state space.

In a simulation study, we generate networks with given degree distributions together with Gaussian data in accordance with their implied conditional independence structures. As a case study we show that 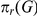 and 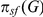 are equally effective at recovering a random network, but that 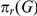 is comparatively ineffective at recovering a scale-free network. In the full simulation study, we confirm that the aforementioned result holds, illustrating our main conclusion: 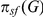 recovers random networks on an equal footing with the 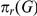, yet surpasses it in recovering scale-free networks. Finally, we illustrate our methodology by analyzing a real gene expression dataset taken from a breast cancer tumor study by [40], showing that in contrast with the random structure prior, the scale-free structure prior recovers hubs, including the estrogen regulator FOXA1 and the zinc transporter SLC39A6, which was previously unrecognized as a hub.

## Methods

### Network Notation

The terms “network” and “graph” are used synonymously throughout this paper. An undirected network 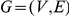 is a mathematical object defined by a set of nodes 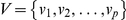 together with a set of undirected edges 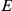 consisting of unordered pairs 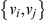 taken from 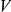, provided that 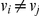. The set of all 
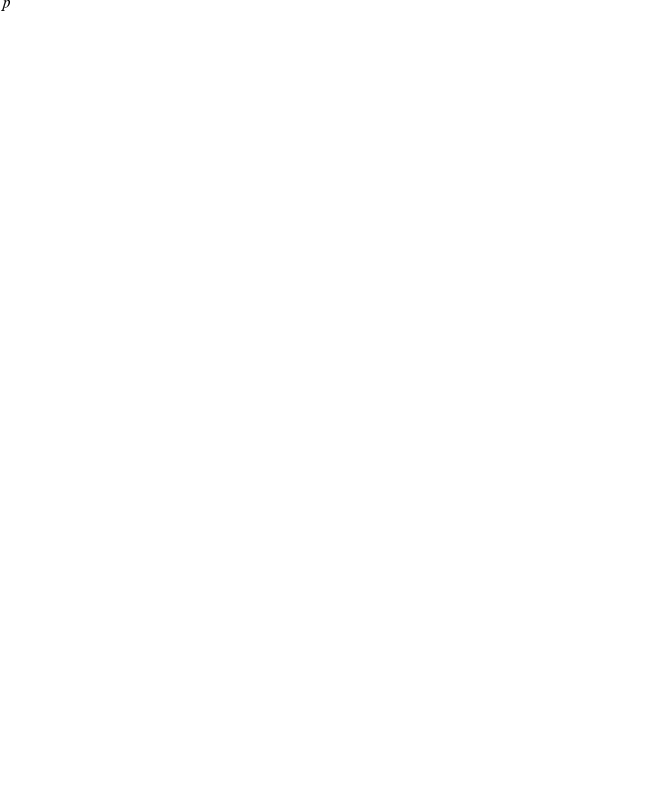
-node, undirected networks is denoted by 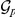. A directed network is defined in an analogous manner, save that the elements of 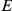 are ordered pairs 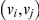 called directed edges; 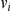 is called the parent and 
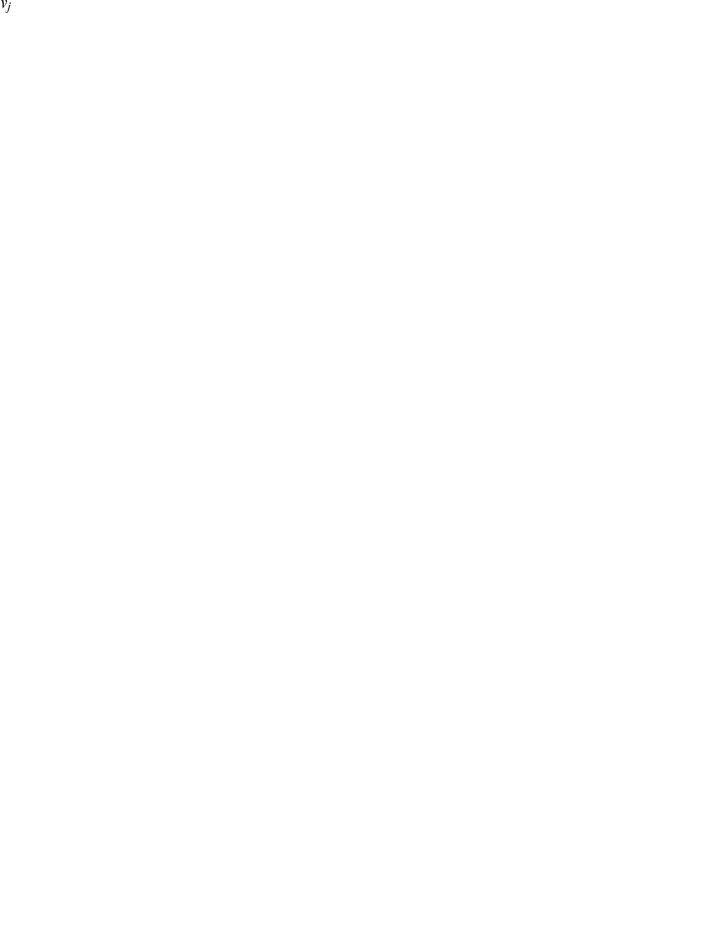
 the child.

**Figure 1:**
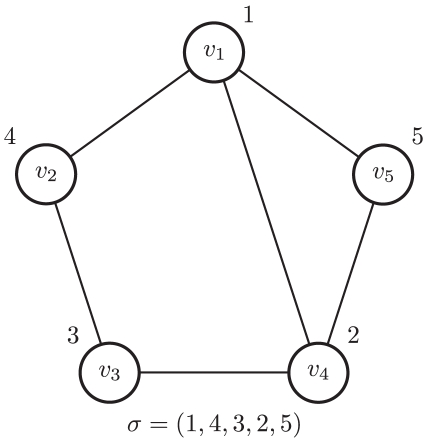
An example of a node labeling. A node labeling, 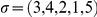, of the nodes of a network with 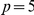 nodes so that, for instance, 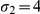 and 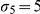.

It should be understood that a network refers to an undirected network, and likewise an edge is to be understood to mean an undirected edge. However, the following definitions are applicable to both undirected and directed networks. An empty network has no edges, that is 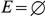, while, in a complete network 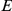 is defined as the cross product 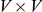. A subnetwork of 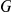 is a network whose node set 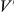 is a subset of 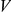, and whose edges are a subset of 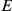 restricted to 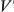. The subnetwork of 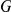 induced by a given subset of nodes 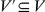 is the subnetwork containing all edges from 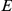 that connect nodes in 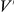. Two nodes are said to be neighbors when they are connected by an edge. And, a network is itself connected when every pair of nodes is connected by a sequence of neighbors. Finally, a node labeling 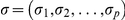 is a permutation of the integers 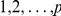, applied to the nodes of 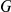 so that each 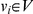 is represented by the integer 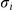; see Figure 1. This node labeling is used later for defining the structure prior.

### Gaussian Graphical Models

In this section we sketch out the theory of GGMs essential to this paper. A detailed overview of the GGM estimation procedures outlined here is described in [25], while [8] is a good starting point for understanding the niche they occupy in the larger context of graphical models.

Let 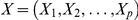 be a 
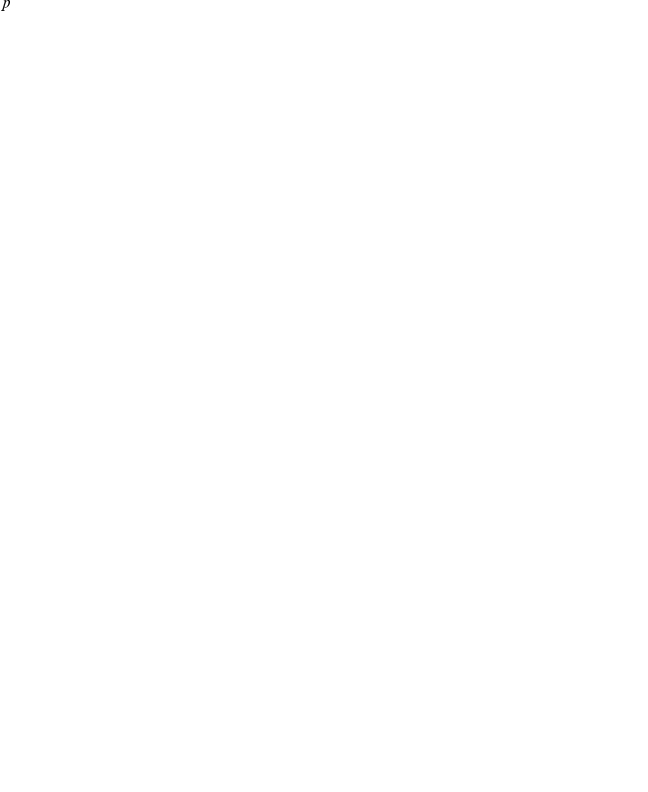
-dimensional Gaussian random vector with zero-mean and positive definite covariance matrix, 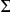. Two random variables 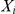 and 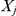 are not conditionally independent given the remaining variables in 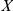 if, and only if, there is a corresponding nonzero entry in the precision matrix, 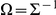 [41]. The conditional independence structure of 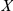 can be represented by a network, 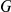, where 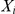 is the value at node 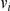 and there is an edge between 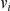 and 
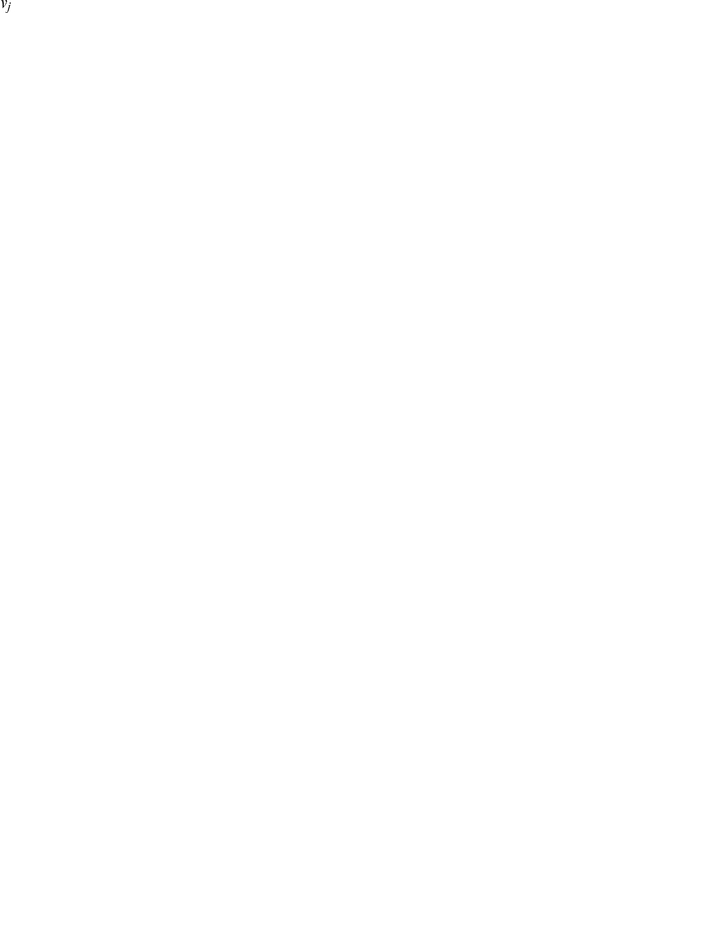
 when 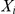 and 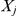 are not conditionally independent. A GGM for 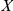 is the family of 
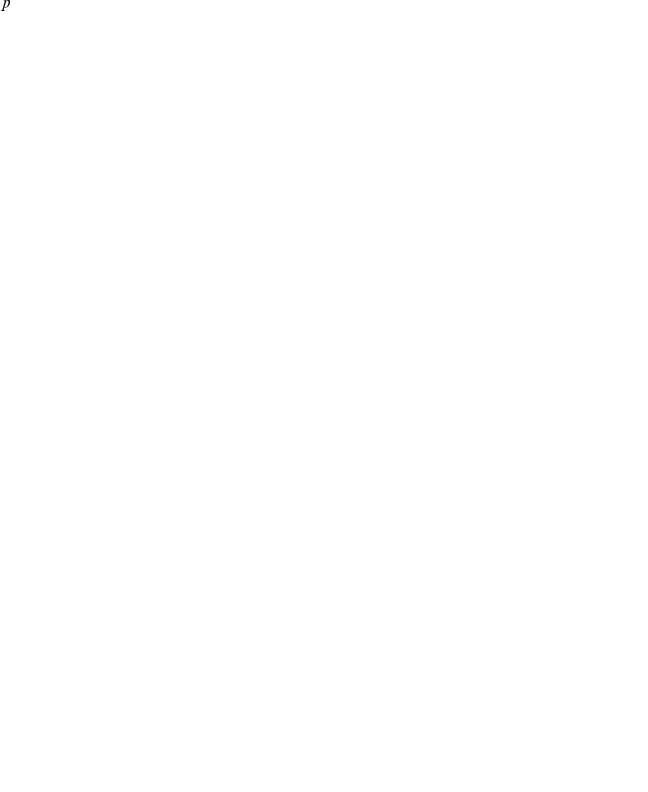
-dimensional Gaussian distributions from 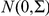, constrained by the structure of 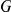.

Fitting a GGM to a given dataset — a task known as covariance selection — amounts to identifying zeros in the estimated precision matrix. In the classical setting when 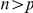, ensuring that 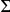 is positive definite, this is typically accomplished by inverting the estimated covariance matrix and then applying statistical tests to identify any entries significantly different from zero [4]. With genomic data, however, “small 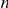, large 
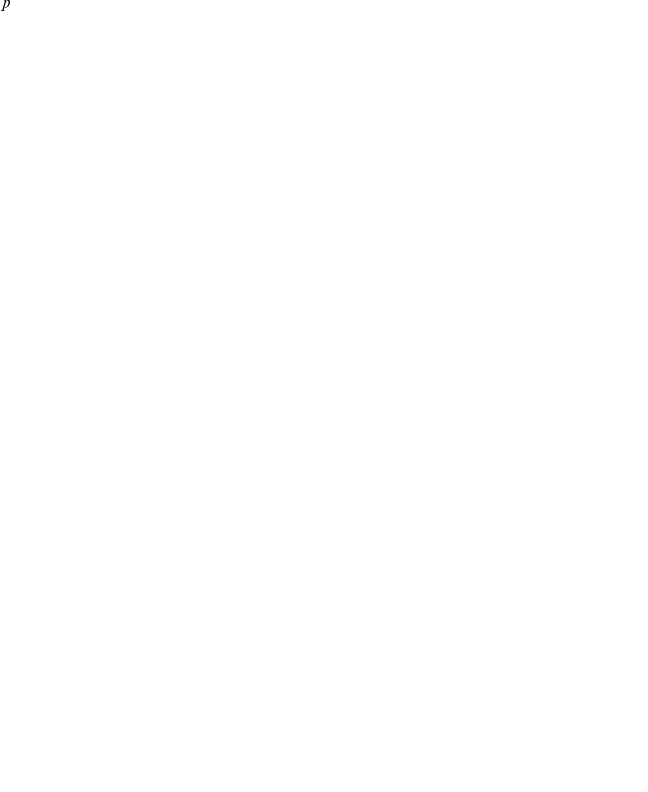
” is the norm and, consequently, 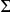 will not generally be invertible.

This problem can be addressed in one of two ways. One way calls for restricting inference to pairwise independencies conditioned on fewer than all 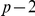 remaining random variables. A relevance network, for example, is constructed by estimating the pairwise correlations between all random variables, connecting any pair with correlation exceeding a specified cutoff value [9]. A related approach goes one step beyond a relevance network by estimating a GAN based on not only marginal but also first-order conditional independencies [11].

A more ambitious approach is to compute satisfactory small sample estimates for 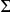 and 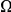 using Bayesian methods. Empirical Bayesian solutions are exemplified by shrinkage estimates [21] and sparsity encouraging lasso regression estimates [22]. Meanwhile, the full Bayesian scheme of [42] works by marginalizing over 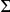 to compute the likelihood term in (1), using a prior that constrains elements of the precision matrix to zero depending on 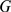:
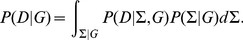
(2)The term 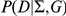 is multivariate Gaussian, while the prior 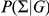 is hyper-inverse Wishart with hyperparameters 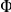, a positive definite dispersion matrix, and 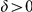, a degrees of freedom parameter. Jones et. al [25] advise a small value for 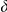 as a reflection of ignorance, and take 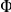 as the diagonal matrix 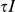, which assumes that the underlying Gaussian variables have common variance. A consequence of this assignment is that 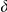 can be used to specify 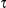 by making use of the fact that the marginal prior mode for each variance term is 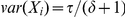.

GGM theory comes equipped with powerful techniques for computing the likelihood function when the underlying network is decomposable. Roughly speaking, a decomposable network can be broken down into distinguished subsets of nodes called maximal cliques. A clique is a subset of nodes whose induced subgraph is complete, and is called maximal when it is not contained within a larger complete subgraph. Computing the likelihood for a subset of 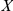 corresponding to a maximal clique is particularly tractable because the density is just an unrestricted multivariate Gaussian. Hence, when a network is decomposable, the evaluation of the likelihood term in (1) reduces to the computation of many likelihoods of smaller dimension [43]. We will return to these issues in the section on our MCMC implementation.

### The Static Model

A network model is a stochastic algorithm for generating networks that may depend on a vector of parameters, 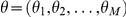. Associated with any model is a probability distribution, assigning a probability 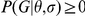 to each 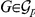, where 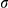 is a node labeling of 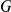.

The static network model [39] works by first assigning a weight 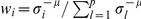 to each node 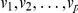 where 
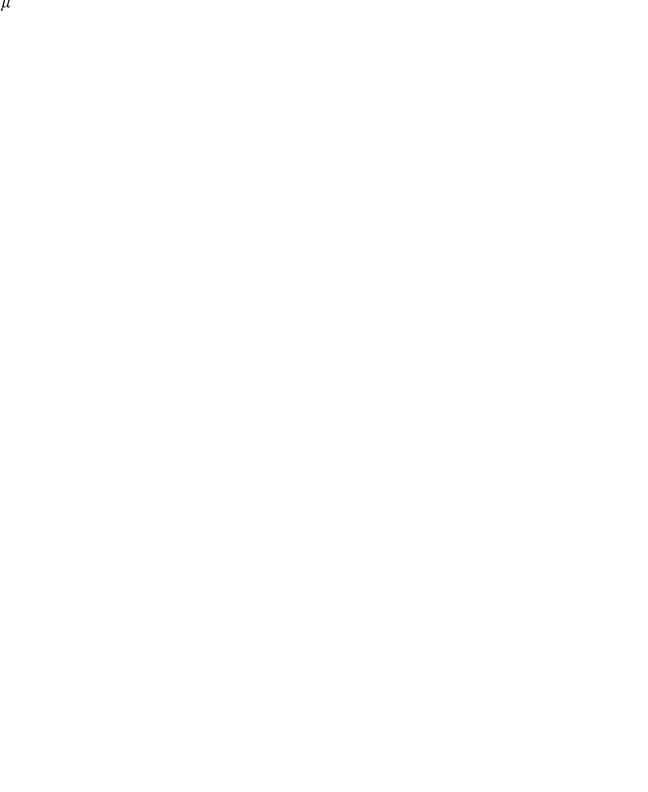
, the Zipf exponent, is a tunable parameter in 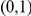. To generate a network, 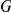, the following step is repeated 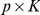 (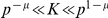) times: select nodes 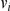 and 
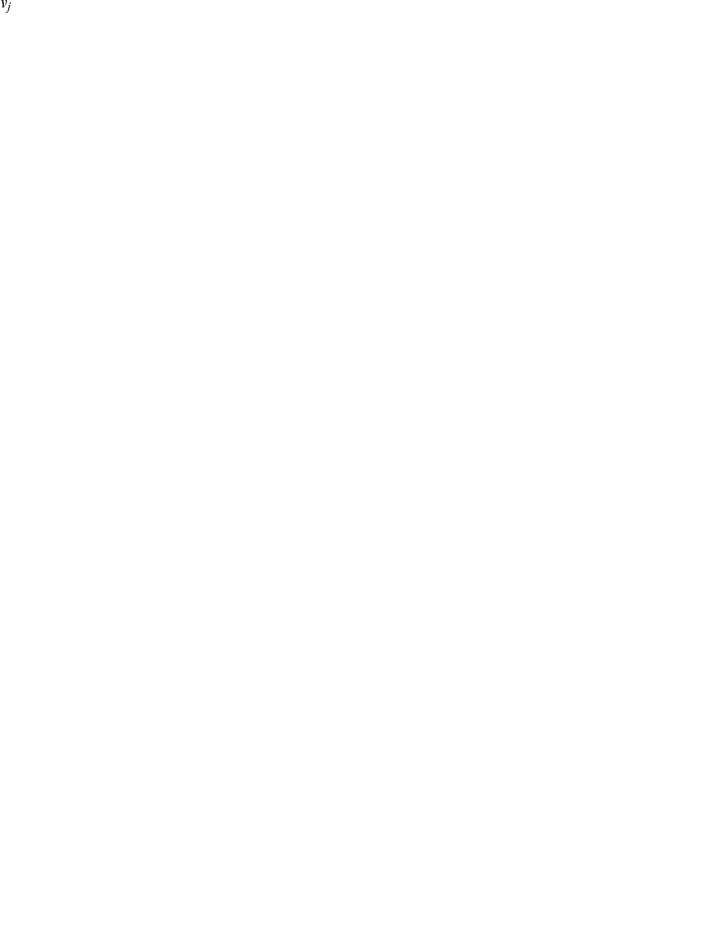
 with probabilities 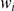 and 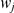 and connect them with an edge, unless 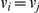 or 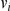 and 
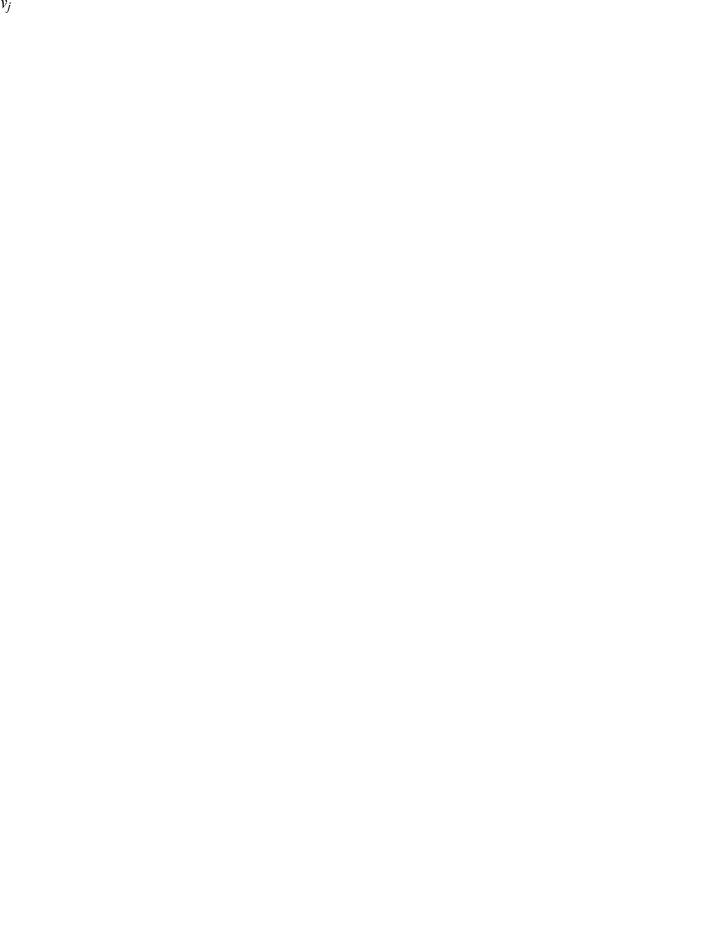
 are already connected, in which case no edge is added to the network. The overall model parameter is 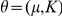.

In order to work out the functional form of the degree distribution, it is enough to notice that, on average, each node acquires edges in proportion to its weight. Supposing for a moment that 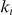 denotes the degree of node 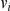, we may write this as 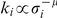. The probability distribution over the 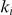
's is known as Zipf's law, and it has been shown to be equivalent to a power-law degree distribution with 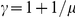 [44]. It follows that the static model generates networks that follow a power-law with 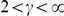 depending on the choice of 
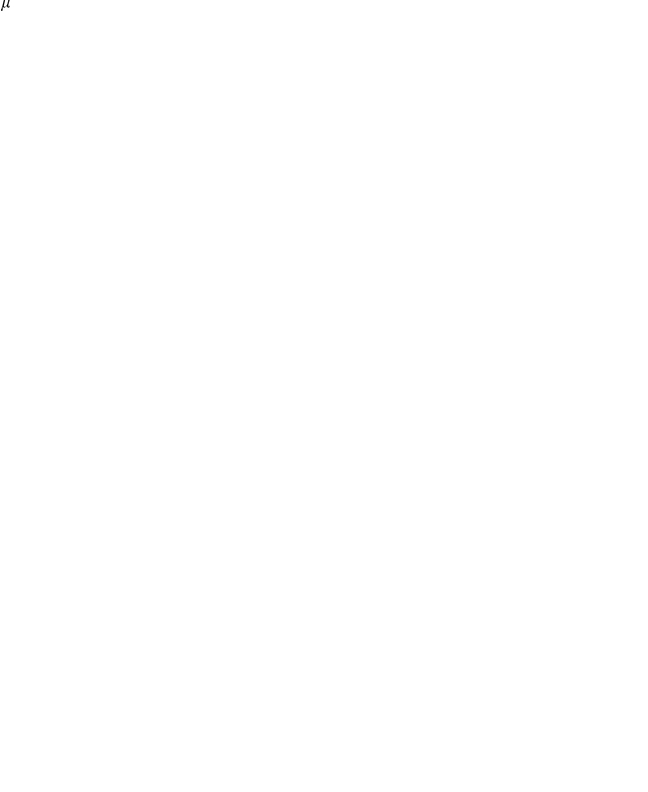
. A rigorous derivation of the power-law appears in a comprehensive analysis of the static model by Lee et al. [45]. In the case when 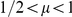, the exponent, 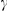, lies between 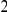 and 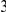, which is the most interesting range of values from the point of view of scale-free architecture. In contrast, for values of 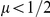, which corresponds to 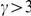, the tail of 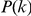 is less pronounced. In the limit of 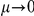, or equivalently 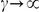, each weight 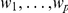 tends to 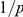, resulting in the ER model with edge inclusion probability 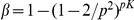. To be clear, the static model actually includes the ER model as a special case.

A formula for the probability of a network is provided in the same analysis. The probability that nodes 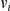 and 
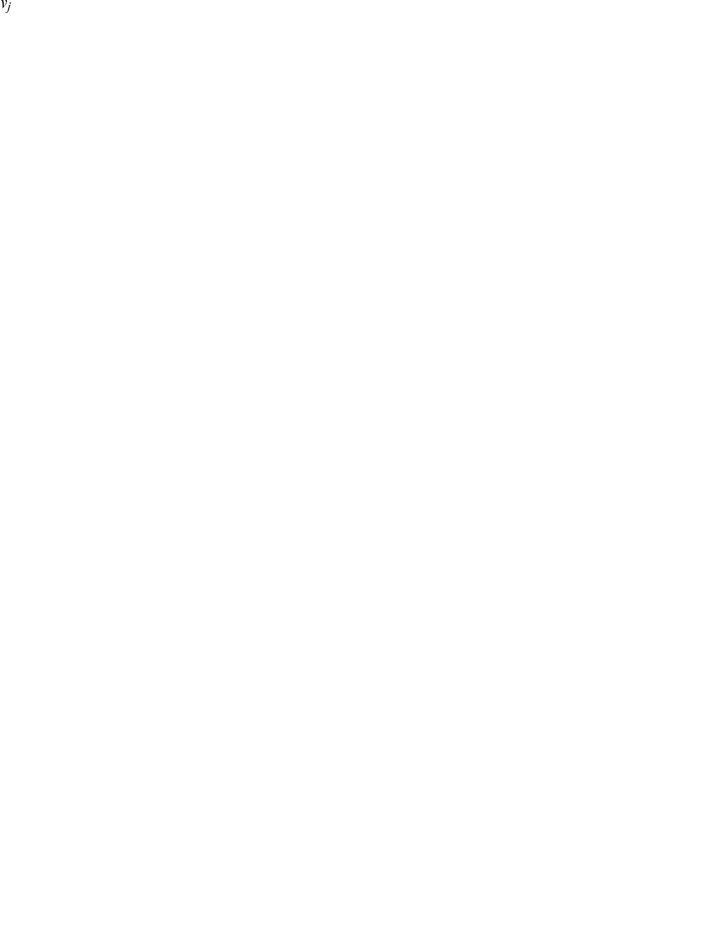
 are connected in the final network is 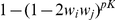, which is well-approximated by 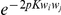 when 
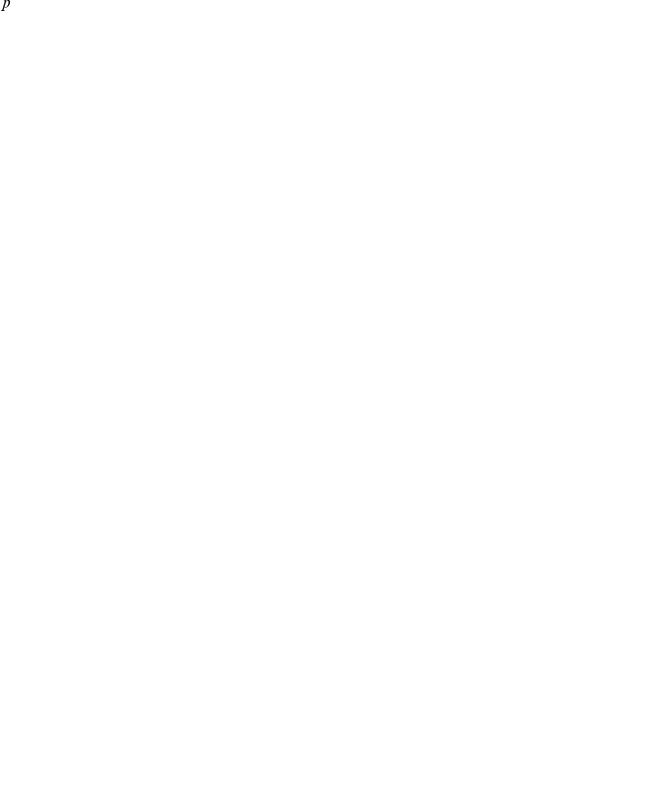
 is large. The probability of a network, then, is given by overall product of the edge inclusion probabilities
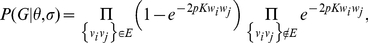
(3)assuming independence.

### A Scale-Free Structure Prior

The structure prior is generically defined as
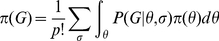
(4)where 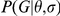 is the probability of a 
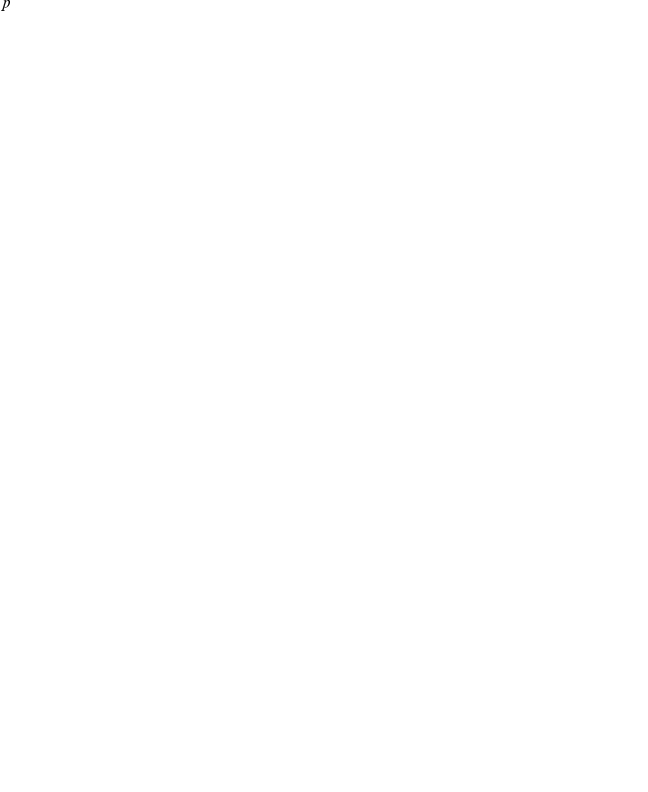
-node network, 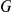, under a certain network model given a vector of parameters, 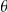, and a node labeling, 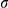; the summation is over all permutations of 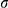. It is obvious from the definition that 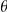 and 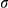 are hyperparameters and must be dealt with accordingly. In the case of 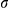, each one of the 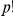 possible node labeling assignments is alloted uniform weight. In our work, we additionally impose that 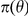 is a uniform prior, leaving the details to be described below within the contexts of specific network models.

The simplest means of dealing with uncertainty about graphical structures is to assign uniform weight either to each 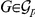, that is, 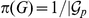, or to the subspace of decomposable networks [43]. This approach is in fact a special case of the probability of a network under the ER network model when 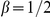. A related approach uses a prior that distributes probability mass uniformly according to the number of edges as opposed to individual networks [42]. More recently, the ER model has been explicitly employed as a structure prior [24], [25]. The random structure prior, 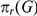, is formally defined by
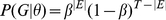
(5)where 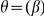 and 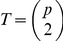 is the number of possible edges. A node labeling would be superfluous due to symmetry. To foster sparsity 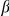 may be fixed at 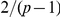 so that the expected number of edges comes out to be 
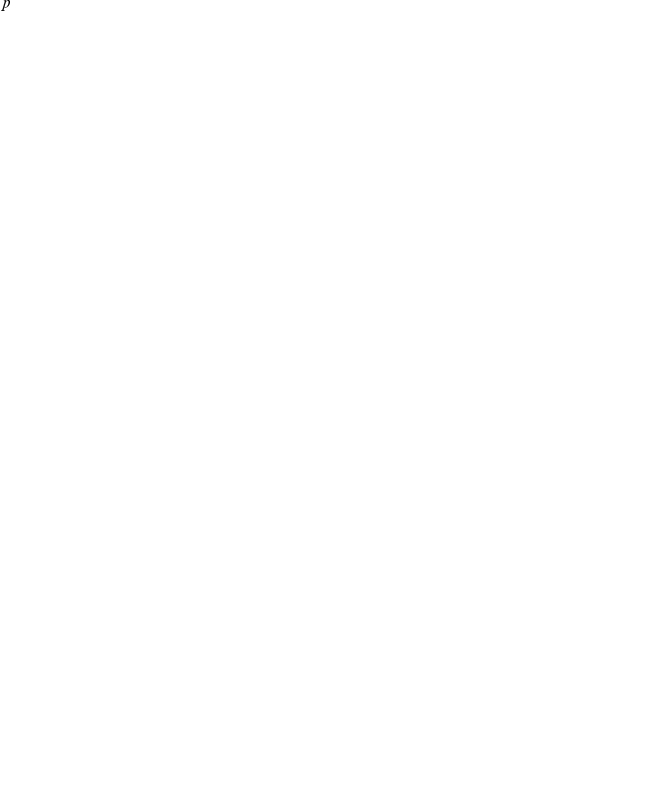
 when (5) is taken over all networks. Although strictly speaking the value will be somewhat lower in the decomposable case [25]. The approach taken in this paper goes one step further by simply taking 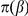 as uniform over the unit interval.

As explained above, the static model with parameter 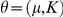 is a generalization of the ER model that is accommodating to scale-free topologies. We define the the scale-free structure prior, 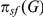, according to the probability of a network under (3). The static model has two parameters, 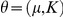, and they are not exactly independent as the domain of 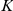 is a function of 
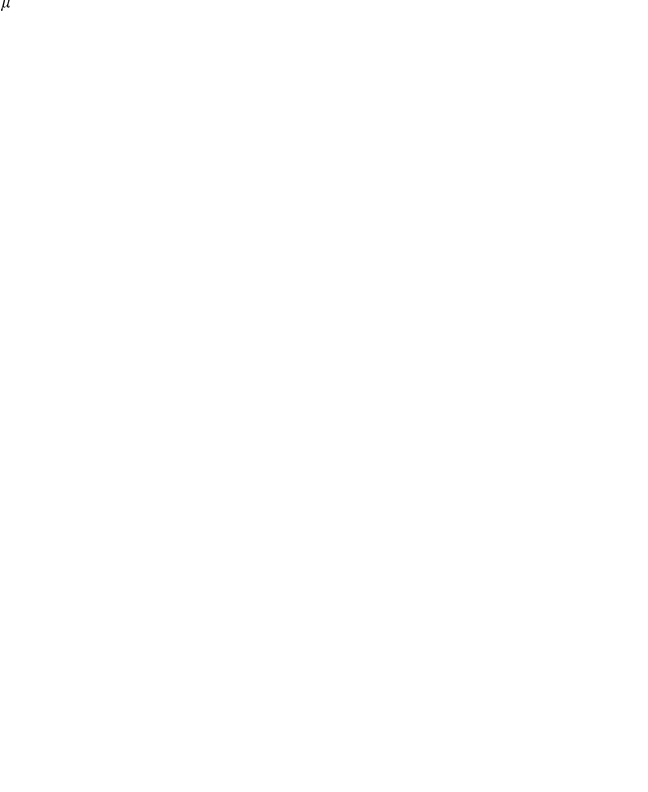
. This means that the prior 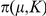 must actually be treated as the product of 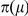 and 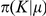. We take each term to be uniform over its respective domain.

### MCMC Implementation

MCMC algorithms are commonly used for sampling from high-dimensional probability distributions such as those encountered in modern bioinformatic applications [46], [47], [48]. In this section, we describe a Metropolis-Hastings sampling scheme for updating the state variables 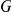, 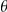, and 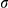. Our main interest is in inference for the posterior 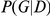. We take the approach of estimating the target distribution
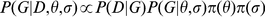
(6)with 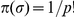 and then marginalize over 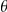 and 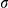 to obtain 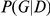. In the process, any 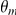 (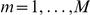) or 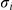 (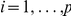) can be estimated from a histogram of values, constructed from an MCMC chain. While methodology for sampling from 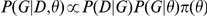 is well-established for GGMs [43], [42], [25], the concept of including 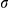 as a state space variable is new to our work. In principle, it is possible to marginalize over all permutations of 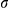 at each step, that is, 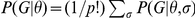. This approach, however, quickly becomes unfeasible as the number of nodes becomes large. What is more, very few assignments for 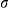 actually capture scale-free network structure, making the marginalization difficult to estimate by random sampling. Instead, we include 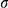 in the MCMC directly. We describe a Metropolis-Hastings sampler for 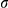 below, and provide an implementation in C computer code for decomposable GGMs, built largely on the work of [25].

### Metropolis-Hastings Sampler

#### Updating 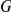


**Figure 2:**
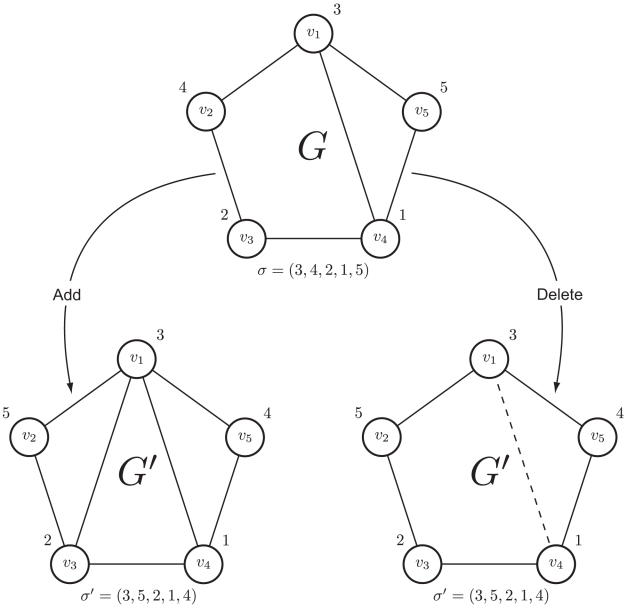
An example of the Metropolis-Hastings transition step. The current network, 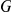, with node labeling 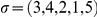 is updated to a proposed network, 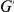, by adding/deleting a single edge to/from 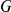 at random. This picture shows two possible ways for 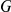 to be updated. In one instance, a new edge is added between 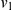 and 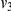 to obtain 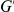, while the other 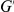 is obtained by deleting the the edge between 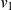 and 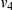. As for the proposed node labeling, an integer 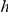, in this example 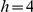, is selected randomly from 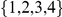. From there the node labels 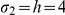 and 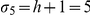 are swapped to get 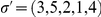.

The space of decomposable graphs can be traversed by adding or deleting a single edge in the transition from a current network, 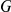, to a proposed network, 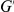 [49]. In an arrangement of this sort, 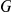 and 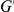 will have nearly identical maximal cliques, leading to extensive cancellation in the likelihood ratio 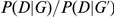 [42]. This coupled with the closed form expressions for (2) in the decomposable case, results in considerable computational savings in comparison with the same computations for non-decomposable models. However, in the transition from 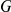 to 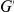, special care is required to preserve decomposability. To that end, a theorem of [43] provides easily verifiable, necessary and sufficient conditions to determine whether or not a network is decomposable. In their implementation [25], a transition is accomplished by first deciding to either add or delete an edge to 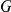 by the flip of a coin. Next the appropriate move is made at random to obtain 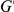 as shown in Figure 2. If 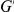 happens to be non-decomposable, then it is rejected outright.

#### Updating 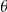


Each hyperparameter 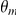 (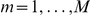) is updated as follows: select a value for 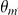 uniformly from 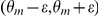 for a given step size 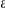, rejecting when 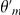 falls outside its domain 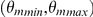.

#### Updating 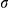


In order to obtain 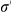 we select an integer 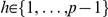 at random, find nodes 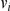 and 
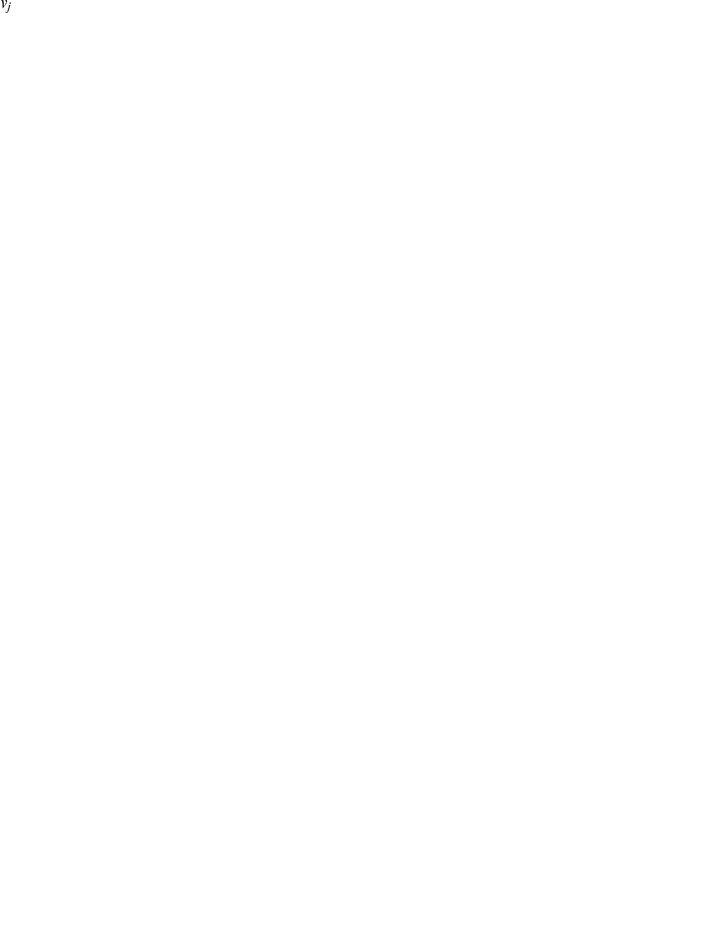
 such that 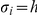 and 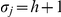, and then exchange the values of 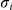 and 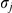; see Figure 2.

### Network and Parameter Estimation

#### Estimating 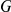


An MCMC sample of the posterior (1) becomes increasingly threadbare as the number of variables grow, so much so that the frequency of a network in a chain is an inadequate approximation to its true probability, even for problems of moderate dimension. So too for the maximum posterior network — the single most probable network in a chain — unless its probability mass dominates a possibly multi-modal landscape, comprising a near-infinity of alternative models, its status as a representative estimator is questionable [50]. This is even more important in our implementation, as we carry the model parameters through the computation. Alternatively, a more representative estimator can be pursued by exploiting marginal probabilities of edge inclusion, which do reflect posterior density. We took our estimated network to be the network of all edges in the sample with marginal probability greater than 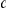, which we denote by 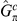; the subscript 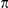 denotes the structure prior.

#### Estimating 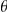


Let 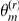 denote the 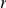
'th value of 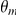 (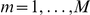) in an MCMC chain of length 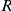. 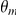 is estimated by averaging over the values in an MCMC sample so that 
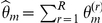
.

## Results

### Simulation Design

We carried out a simulation study in order to evaluate the relative performance of the random and scale-free structure priors. In our experiments, we generated trees invested with a variety degree distributions that can be thought of as falling along a spectrum ranging from binomial to scale-free on through to more extreme heavy-tail forms, called crumple trees, culminating finally with a star tree. For each tree, we generated multivariate Gaussian data under the assumption that a tree represents the true underlying conditional independence structure of a GGM. We then ran our Metropolis-Hastings sampler for both structure priors in an effort to recover each true tree from the data.

#### Data generation

In order to simulate trees we more or less relied on the stochastic algorithm of [51]. Their approach rests on specifying a formula for the degree distribution, 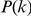, for a 
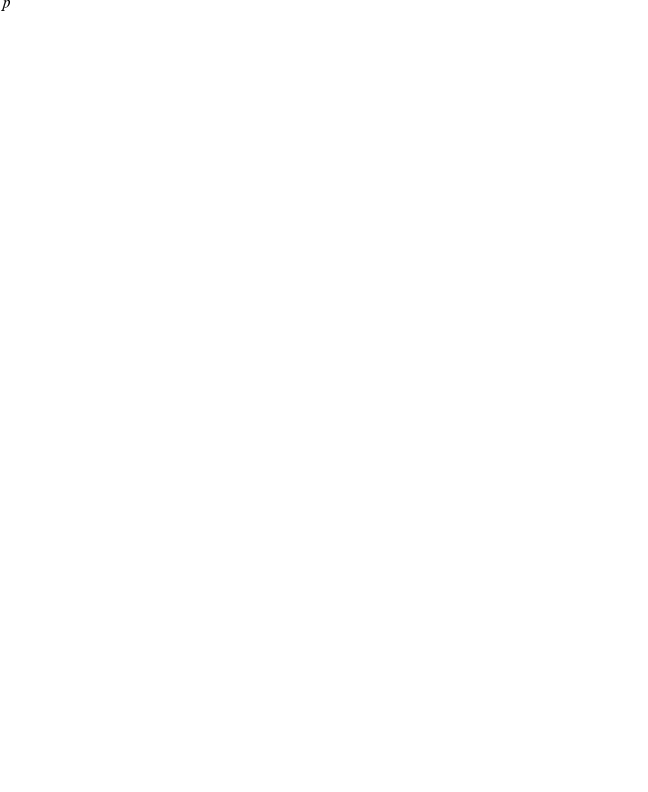
-node connected tree. Then, roughly speaking, they use MCMC to draw a tree that is maximally random under 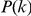.

The reason for restricting our simulation to trees is that data satisfying their implied conditional independence structures can be generated by a simple iterative procedure. With this end in mind, it is convenient to imagine the edges as being directed according to index so that an edge from 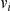 to 
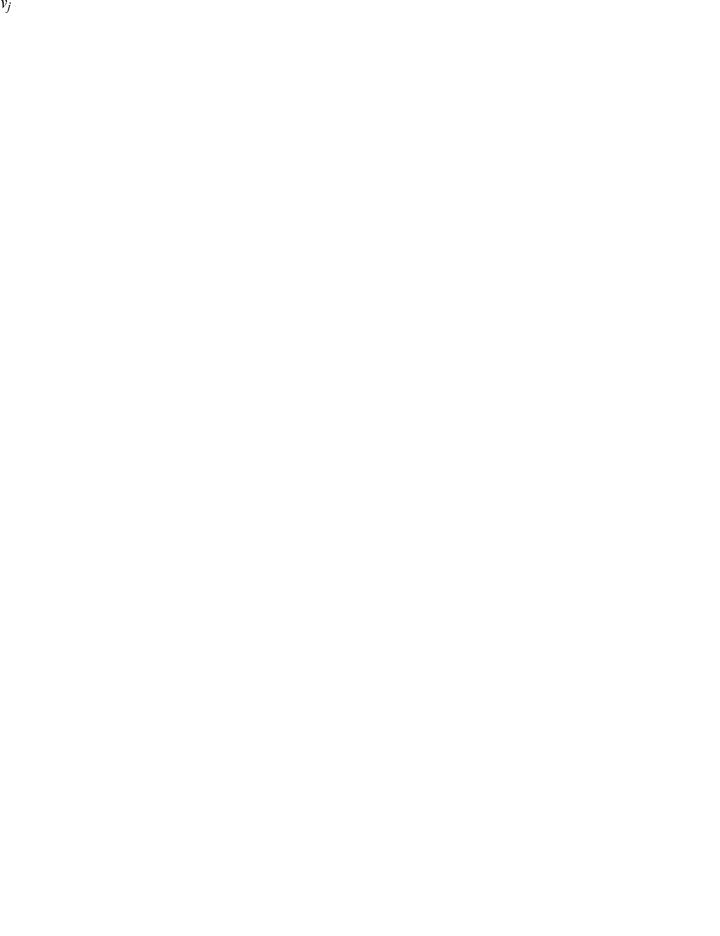
 implies that 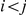. The procedure begins with simulating 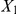, which is identified with node 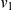, as a standard normal random variable, 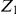. Next, any 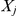 corresponding to a child of 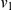 is simulated as 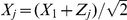. The step 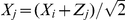 is then repeated from parent 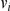 to child 
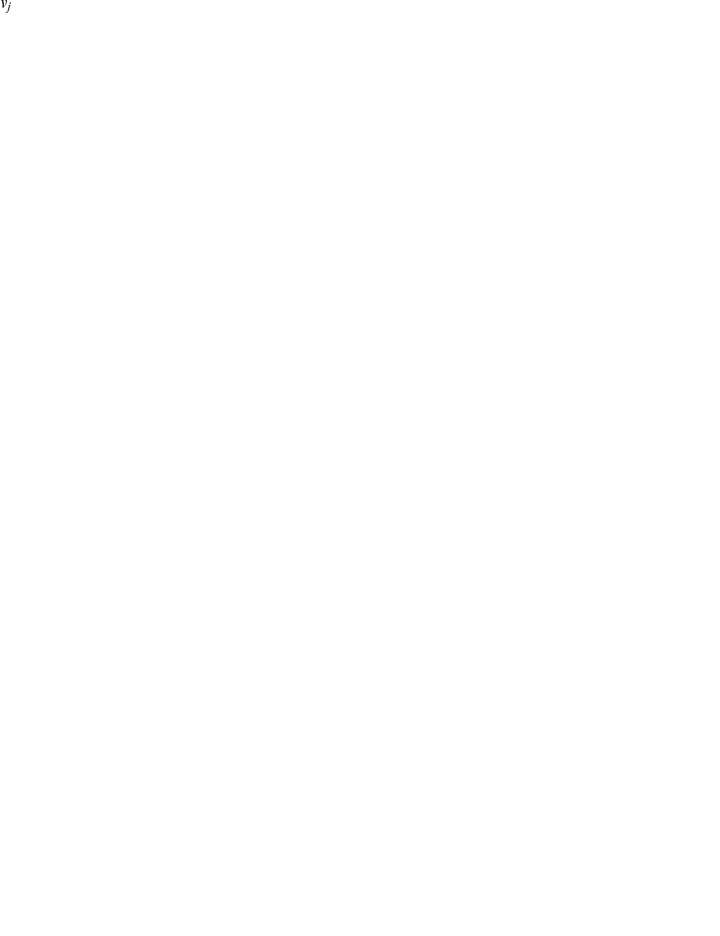
 until all nodes have been reached. The scaling factor, 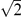, ensures that each 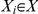 has unit variance.

#### Performance measures

Let 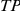 (true positive) denote the number of edges correctly identified by the estimated network with 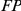 (false positive), 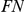 (false negative), and 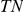 (true negative) defined similarly. The positive predictive value, 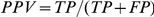, and the sensitivity, 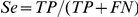, are reported for each estimated network. While it is often customary to include specificity, 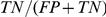, along with 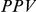 and 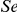, its conspicuous absence here is for good reason. Since GANs are sparse, 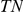 is sure to be very large in comparison to 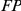. As a result, even a moderate change to 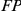 will have little influence on the specificity, making this an unsuitable measure of performance.

### Simulated Example

This section serves as a prelude to an extensive simulation study, illustrating our methodology by means of a simple example. Specifically, we set 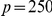 and generated a binomial tree and a scale-free tree with 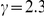, and then simulated an 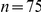 observation dataset from each. In each case, we attempted to recover the true tree from the scaled dataset using our Metropolis-Hastings sampler implemented with 1) 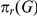, and 2) 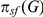.

For each chain, the Metropolis-Hastings sampler was run for 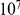 steps after a burn-in of 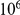, starting from the empty network and identity node labeling. The value for the step size, 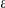, required for updating the hyperparameters was set to 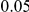 with 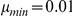. As for the hyper-inverse Wishart parameters, we choose 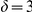 which fixes 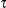 at 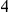 since the data was standardized. The values of the hyperparameters were recorded at every 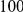
'th step after burn-in. The runtime for the Metropolis-Hastings sampler with 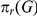 on a dual 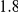 GHz PowerPC G5 processor was 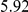
hrs for the binomial tree and 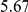
hrs for the scale-free tree. The corresponding runtimes with 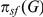 were 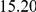
hrs and 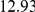
hrs.

The results of the case study are shown in Table 1. In this experiment, our expectation that 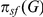 will recover the scale-free tree more accurately than 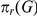 is confirmed. It should also be noted that 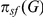 was able to recover a reasonable value for the scale-free exponent, too. Not to mention that it recovered the binomial tree on par with 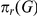, thereby allaying the potential drawback that it would infer a heavy-tailed network, even from binomial data. Remember, this can be explained by the rather large value of 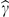. Recall that when 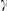 is large, 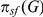 actually approximates 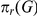. And although it may seem odd that 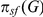 fared slightly better on the binomial tree, the disparity falls within the boundaries of sampling variation. More precisely, we ran the Metropolis-Hastings sampler 10 times for each structure prior, starting each run from a different random seed, and found that the standard deviation of the sensitivity was 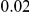 in each case. Finally, we ran the uniform structure prior on both trees, but decided against including the results in Table 1 due to very poor performance.

**Table 1:** Case study.

		Random Structure Prior		Scale-Free Structure Prior		
Topology	True 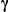	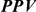	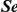	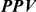	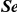	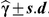
Binomial	—	0.96	0.59	0.96	0.60	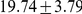
Scale-Free	2.3	0.88	0.54	0.90	0.79	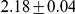

Summary of the networks estimated using 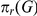 (left) and 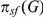 (right) when the true tree topology is binomial in the one case and scale-free with 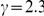 in the other. 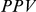 is the positive predictive value and 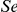 the sensitivity, which is computed in respect to the the number of edges correctly identified by the estimated network. The value 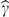 is the estimated scale-free exponent, obtained by averaging over every 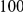
'th value in the MCMC chain.

### Extended Simulation

Table 2 contains the results of our main simulation. In the previous section, we focused on two particular trees: one binomial, the other scale-free. This time we generated 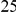 trees (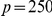) under each model listed in the table together with accompanying datasets of 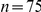 observations. The models listed as scale-free, not including the BA model, and the crumpled one were generated from a two-parameter family of distributions [51]. The parameter setting for generating the crumple trees was 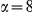 and 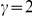. The simulation settings used for each MCMC run are identical to those of the case study. Finally, the values of 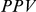, 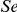, and 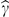 reported in the table are averaged over the 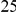 chains. The simulation was run on the supercomputer, Tsubame [52]. The system has a total of 639 Sun Fire ×4600 nodes. Each node has 8 AMD Opteron Dual Core model 880, 2.4GHz cpus.

**Table 2:** Full simulation.

		Random Structure Prior		Scale-Free Structure Prior		
Topology	True 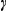	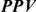	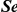	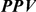	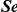	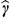
Binomial	—	0.96	0.59	0.96	0.60	18.70
Scale-Free (BA)	3.0	0.95	0.58	0.94	0.65	2.49
Scale-Free	2.5	0.93	0.56	0.93	0.62	2.35
Scale-Free	2.3	0.86	0.49	0.90	0.71	2.18
Crumple	—	0.76	0.38	0.90	0.80	2.10
Star	—	0.63	0.30	0.90	0.86	2.10

Summary of the networks estimated using 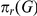 (left) and 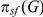 (right) for a variety of topologies. A total of 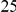 trees of were generated for each kind of topology; each has 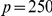 nodes with an accompanying 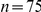 observation dataset. 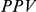 is the positive predictive value and 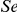 the sensitivity, which are computed according the the number of edges correctly identified by the estimated network. 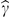 is the estimated scale-free exponent. The values in the table are averaged over the 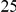 MCMC runs.

Just as with the simulated example, 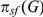 recovers the binomial trees equally as well as 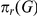. In fact, the 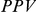 agreed to two decimal places, while the 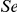 was actually a little higher under the scale-free structure prior. This slight discrepancy can be accounted for by noting that the standard deviation of 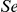 was 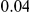 for both priors. Also as expected, the more heavy-tailed the underlying trees become, the more 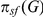 outperforms 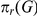. The difference becomes huge in the extreme case of a star tree. Moreover, 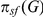 demonstrated the ability to roughly recover the scale-free exponent of the underlying tree.

### Real Data Example

We demonstrate our methodology on a subset of the gene expression data from a breast cancer study by [40] that was originally analyzed in [25]. The dataset (Dataset S1) consists of expression profiles for 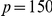 genes related to the estrogen receptor gene ESR1 (also known as ER-alpha) derived from 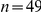 tumor samples. This gene is an estrogen-activated transcription factor key to the proliferation of cancerous cells that is found to be overexpressed in luminal type A and B breast cancers. The overall level of ESR1 expression is higher in type A than in type B with the former correlating with better prognosis [53].

The Metropolis-Hastings sampler was run on the standardized data with both the random structure prior and its scale-free counterpart, yielding the corresponding GANs 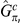 and 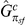. For comparison's sake, the edge inclusion threshold, 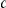, was tuned for each run so that the resulting GAN comprised exactly 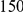 edges; the value of 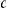 is 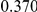 for 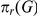 and 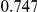 for 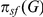. In both cases, the Metropolis-Hastings sampler was started from the empty network with identity node labeling and 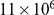 iterations were run with the first 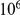 discarded as burn-in. The hyperparameter assignments were identical to those of the simulated examples. The runtime on a dual 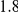 GHz PowerPC G5 processor was 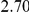
hrs with 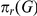 and 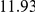
hrs with 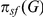.

At this stage, comparing the performance of the scale-free structure prior in a broader context is of key importance. To this end, we used the software packages ARACNE [54], [55] and BANJO [23] to analyze the gene expression data as well. ARACNE constructs a relevance network based on estimated mutual information between all pairs genes, but there is a twist. After a relevance network is inferred by connecting any pair of genes with mutual information greater than a certain cutoff value, some edges suspected to represent indirect interactions are eliminated using the data processing inequality principle. We chose the cutoff value to be 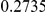 so that the number of estimated edges was 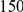, while all other program arguments were set at their default values. The code itself was run in a matter of minutes. BANJO, on the other hand, constructs a Bayesian network from discrete data using a heuristic search strategy to explore the space of 
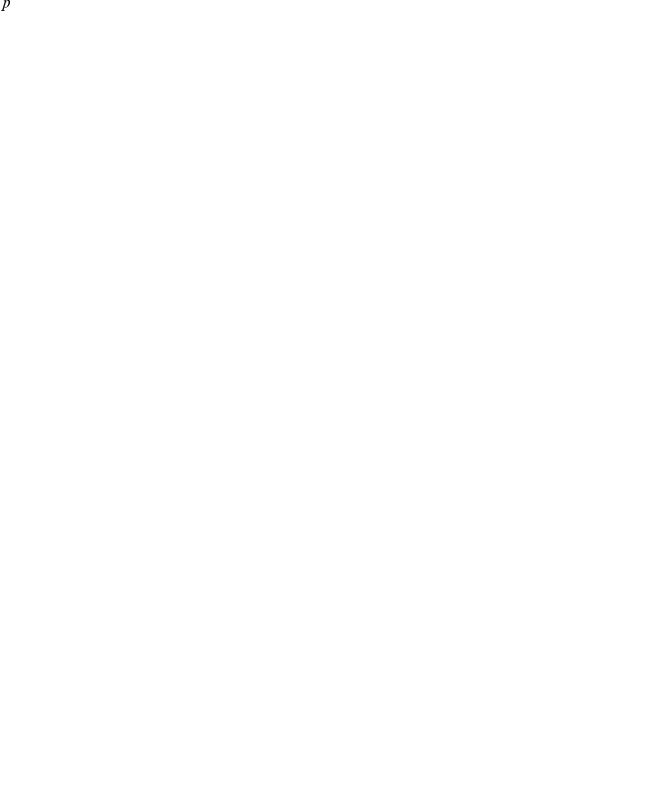
-node, directed networks without cycles. Each network happened upon in the search is ranked using a Bayesian Dirichlet equivalence scoring metric. We discretized the data into three categories and limited the number of parents any given node may have to 10. Once again, all other program arguments were set at their default values. The estimated network, which was found to have 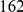 directed edges, was the highest scoring network after running BANJO for 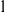
hr.

**Figure 3:**
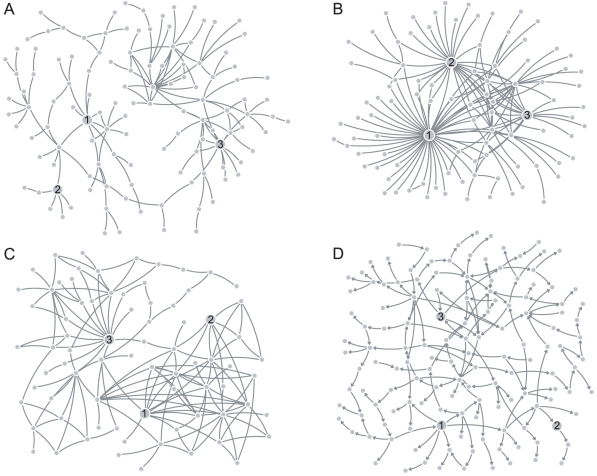
Estimated networks for the breast cancer expression dataset. (A) The gene association network estimated with random structure prior, 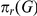, and (B) the gene association network estimated with the scale-free structure prior, 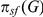. (C) The relevance network estimated with ARACNE, and (D) the Bayesian network estimated with BANJO. The labeled nodes are the largest hubs as identified by 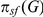 (1:FOXA1, 2:SLC39A6, 3:E2F3). For ease of visualization, only the largest connected subnetworks are displayed.

Figures 3(A) and (B) show the GANs estimated with 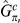 and 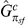. The latter exhibits clear hubs, supporting the view that a gene regulatory network consists of a small minority of hub genes with the vast majority of genes engaged in a small number of interactions. By contrast, the topology of 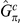 is relatively decentralized with no single gene dominating the network. Additionally, the estimated value of exponent 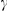 in the static model was 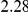, in line with findings in the literature for gene regulatory networks [31]. Turning now to Figures 3(C) and (D), it is interesting to see that the topology of the relevance network echoes that of the GAN inferred using the scale-free structure prior. The same can be said for the Bayesian network and the random structure prior GAN. Of course, a more exquisite experimental technique is the only sure-fire way to validate the individual regulatory interactions suggested by these graphical models. These results, however, are telltale in one respect. In a study comparing different reconstruction methods on simulated data [56], it was reported that BANJO performs well only when 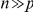, while ARACNE shows good performance even when 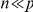.

**Figure 4:**
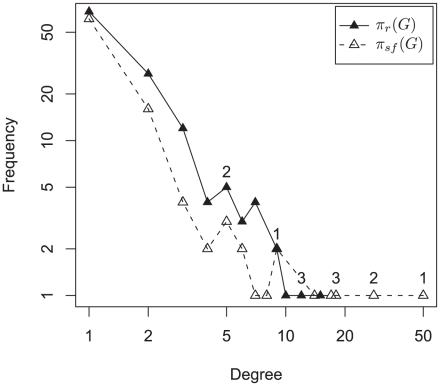
Degree distributions of the gene association networks. The labels 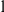, 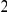, and 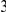 indicate the locations of the genes FOXA1, SLC39A6, and E2F3, respectively. The plots are on a 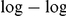 scale.

The topological dissimilarity between the two GANs is again made evident by a visual inspection of their degree distributions, plotted in Figure 4. The most abundantly connected node in 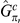 has degree 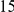, whereas 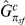 contains four nodes with degree exceeding this value; the largest hubs correspond to the genes FOXA1 (HNF-3A), SLC39A6 (LIV-1), and E2F3 (KIAA0075) and have degree 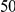, 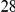, and 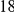, respectively. The main hub FOXA1 is a forkhead box family transcription factor that is necessary for optimum expression of roughly half of all ESR1-regulated genes [57]. In a recent study [58], it was found that FOXA1 is expressed predominantly in luminal type A carcinomas, making it a potential marker of good prognosis. Previously unrecognized as a hub, SLC39A6 functions as a zinc transporter, and was identified in [59] to be highly expressed in ESR1-positive tumors as well as showing a highly significant association with the spread of breast cancer to the lymph nodes. Meanwhile, E2F3 is a transcription factor that has been shown to regulate numerous genes involved in cell cycle progression [60].

**Figure 5:**
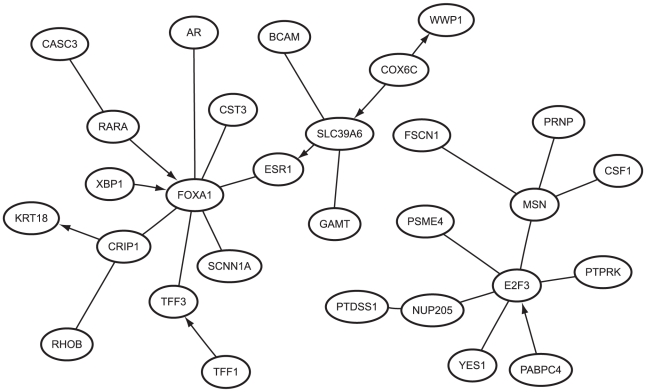
Gene interactions identified by all methods. A subnetwork of the gene interactions involved with the estrogen receptor gene, ESR1, that were commonly identified by the random structure prior, 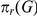, the scale-free structure prior, 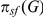, and the relevance network (estimated with ARACNE). The directed edges indicate the gene interactions on which the Bayesian network (estimated with BANJO) agreed with the other three methods.

Finally, both GANs agreed with the relevance network on some established regulatory interactions as can be seen in Figure 5. For instance, FOXA1 is connected to AR (androgen receptor), which is known to regulate estrogen receptor expression [61]. FOXA1 has also been shown to play a direct role in the transcription of the TFF1 (pS2) gene [62], and our work agrees with [24] on the role of TFF3 (ITF) as an intermediary. By contrast, the Bayesian network agreed on very few of these interactions. Part of the explanation is likely to rest in using the maximum posterior network as the estimated network. As we drew attention to in the section Network and Parameter Estimation, a single network of high posterior probability may be a less representative estimator than an network consisting of edges that occur with high frequency in an MCMC chain. Another possible contributing factor is that the number of observations was insufficient for BANJO, but what is also unclear is the extent to which discretizing the expression data affected the quality of the inference.

## Discussion

The main purpose of this paper has been to introduce a scale-free structure prior, 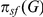, for graphical models with a view toward the inference of large-scale GANs from datasets consisting of few observations, 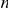, for a comparatively large number of variables, 
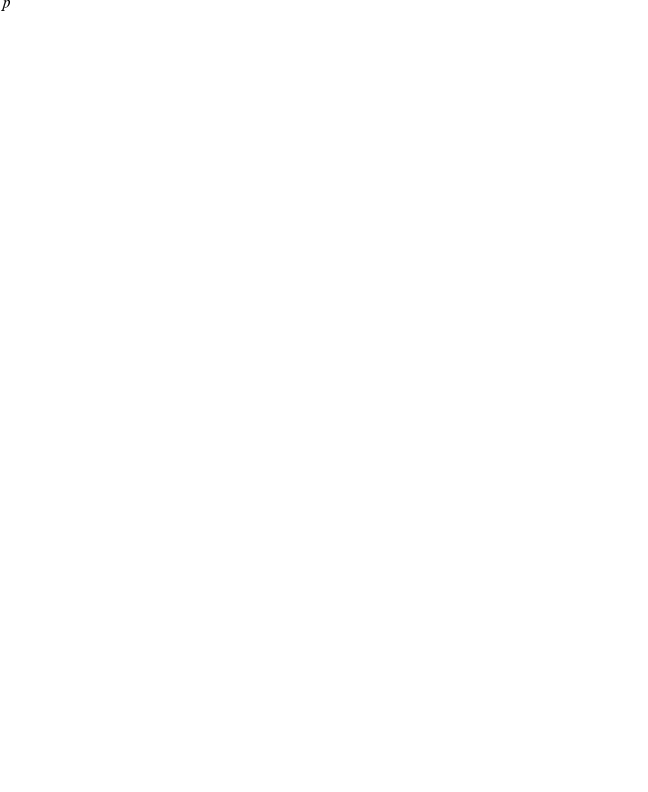
. It is important to point out that the true network need not follow a power-law in order for the scale-free prior to be applicable; rather, 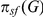 is a convenient distribution that can account for heavy-tailed degree distributions — a crucial limitation of the random structure prior. That said, we have shown in simulated examples that 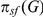 performs markedly better than the random structure prior at recovering networks characterized by heavy-tailed degree distributions. What is more, 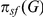 proved versatile enough to recover random networks on par with 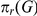 itself. Above all, our analysis of the breast cancer expression data illustrates the practical value of the scale-free structure prior as an instrument to aid in the identification of candidate hub genes with the potential to direct the hypotheses of molecular biologists, and thus drive future experiments.

A node labeling 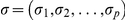, that is, a permutation of the integers 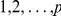 applied to the nodes of 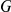 so that each 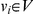 is represented by the integer 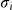, is an essential prerequisite for any MCMC implementation of the scale-free structure prior. The reason is that the scale-free network model underlying 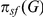, or any other scale-free network model for that matter, is so elaborate that the nodes are not interchangeable in regard to computing the probability of 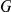. And, although easily overshadowed by 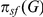 itself, our new Metropolis-Hastings sampler for 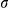 is an innovative contribution in its own right. Our sampler uses a simple pair swapping strategy for updating 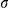, and one future topic of research is to investigate the comparative performance of more ingenious update schemes. More research is also required in order to assesses how accurately 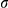 can be estimated.

We take pains to point out that while our implementation is for GGMs, the methodology described here applies to graphical models more generally. For instance, 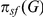 could be applied crudely to Bayesian network inference by simply ignoring edge directionality, or else the underlying static model could be modified to have directed edges. The latter approach raises an interesting consideration: in gene regulatory networks, according to the prevailing wisdom [31], it is actually only the out-degree distribution that follows a power-law. By contrast, the in-degree of a node is usually small and its distribution is better approximated by a sort of restricted exponential function. While this distinction gets blurred when inference is conducted with undirected graphical models, Bayesian networks provide an obvious incentive for taking it into account. Indeed, Bayesian networks may prove to be a more promising area of application because they currently able to handle much larger networks than GGMs [63].

Although the static model is not biologically motivated, it is a defensible choice as an underlying model for 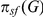 on the grounds that it is a simple model with the potential to describe any network topology; not to mention that it includes the ER model as a limiting case. But there is more, implementing a structure prior based on a growing network model poses some added difficulties because not only will the probability of a network depend on the choice of seed network, but evaluating 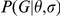 will result in a greater expenditure of computational resources as the edge inclusion probabilities depend on the order in which they were added to the network.

All the same, we implemented two other scale-free structure priors based on growing models; one on the Poisson-growth, preferential attachment model [64], and another on the biologically meaningful duplication model. In the former case, we were able to get away with using a single node as the seed network, and we found that while this prior recovered heavy-tailed networks as well as 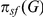, yet it understandably struggled to accurately recover random networks. Meanwhile, the duplication model based structure prior was highly sensitive to the choice of seed network in addition to being unstable due to the complexity of the model. One future avenue of research is to adapt these models, or the MCMC implementation, to be more applicable for use as a prior distributions. The primary motivation for doing so is that the model parameters have biological meaning, and their estimation could prove of independent interest.

The estimation of network model parameters has been an incidental aspect of our work; however, it is related to the quite different problem of fitting network models to known biological networks. Likelihood and likelihood-free methods have been developed [65], [66] in order to fit a hybrid preferential attachment/duplication and divergence model to some protein-protein interaction networks, obtaining estimates of the model parameters. These methodologies assume that the ordering of the nodes in time, that is 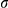, is known, but in most cases this information is unknown. In the future, our Metropolis-Hastings sampler could very well be applied to this problem.

Software is available from the corresponding author upon request.

## Supporting Information

Dataset S1 This file contains the gene expression data that we analyzed in our paper.
(0.05 MB TXT)
